# Restriction of meat, fish, and poultry in omnivores improves mood: A pilot randomized controlled trial

**DOI:** 10.1186/1475-2891-11-9

**Published:** 2012-02-14

**Authors:** Bonnie L Beezhold, Carol S Johnston

**Affiliations:** 1 Nutrition Department, Benedictine University, 5700 College Road, Lisle, Illinois, USA; 2 School of Nutrition and Health Promotion, Arizona State University, 500 N. 3rd Street, Phoenix, AZ, USA

## Abstract

**Background:**

Omnivorous diets are high in arachidonic acid (AA) compared to vegetarian diets. Research shows that high intakes of AA promote changes in brain that can disturb mood. Omnivores who eat fish regularly increase their intakes of eicosapentaenoic acid (EPA) and docosahexaenoic acid (DHA), fats that oppose the negative effects of AA in vivo. In a recent cross-sectional study, omnivores reported significantly worse mood than vegetarians despite higher intakes of EPA and DHA. This study investigated the impact of restricting meat, fish, and poultry on mood.

**Findings:**

Thirty-nine omnivores were randomly assigned to a control group consuming meat, fish, and poultry daily (OMN); a group consuming fish 3-4 times weekly but avoiding meat and poultry (FISH), or a vegetarian group avoiding meat, fish, and poultry (VEG). At baseline and after two weeks, participants completed a food frequency questionnaire, the Profile of Mood States questionnaire and the Depression Anxiety and Stress Scales. After the diet intervention, VEG participants reduced their EPA, DHA, and AA intakes, while FISH participants increased their EPA and DHA intakes. Mood scores were unchanged for OMN or FISH participants, but several mood scores for VEG participants improved significantly after two weeks.

**Conclusions:**

Restricting meat, fish, and poultry improved some domains of short-term mood state in modern omnivores. To our knowledge, this is the first trial to examine the impact of restricting meat, fish, and poultry on mood state in omnivores.

## Introduction

Research evidence has linked long-chain omega-3 (n-3) fatty acid intake to mood [1], an important link since diets can vary greatly in fatty acid content. Fish and shellfish are among the few dietary sources of long-chain n-3 fatty acids, eicosapentaenoic acid (EPA) and docosahexaenoic acid (DHA), whereas diets rich in meat and poultry are high in the potentially neuroinflammatory long-chain omega-6 (n-6) fatty acid, arachidonic acid (AA) [2]. Moreover, in omnivores who consume low amounts of fish, the elevated AA to EPA/DHA ratio in the diet is mirrored in membrane phospholipids, a profile associated with depressive symptoms [3]. Omnivorous diets rich in fish are associated with a lower risk of depressive symptoms [4].Vegetarian diets that restrict meat, fish, and poultry are low in both long-chain n-3 and n-6 fatty acids as compared to omnivorous diets [5], but there is limited data exploring the effects of a vegetarian diet on mental health.

We recently observed that vegetarians reported better mood than omnivores despite their negligible intake of EPA/DHA [6]; these data suggest that the dietary ratio of long-chain fatty acids may have an effect on mood. This pilot trial examined the mood effects of removing meat, fish, and poultry from the diet of healthy omnivores. We hypothesized that omnivores who avoided intake of meat, fish, and poultry would report better mood than control omnivores who continued to eat meat, fish, and poultry daily.

## Methods

Our study design was a parallel arm, two-week randomized controlled trial. Adult men and women who reported consuming meat and/or poultry at least once a day were recruited for this trial, which was announced as an investigation of the role of protein in brain function. Individuals who were pregnant or lactating, drank more than 12oz per day of alcoholic beverages, diagnosed with a mental disorder, or used substances that modulate mood were excluded. Participants (n = 39) provided written informed consent, and the study was approved by the Institutional Review Board at Arizona State University.

A general health history was completed at baseline; at baseline and at trial completion, dietary fatty acid intake and mood were assessed using a validated food frequency questionnaire [7] and two validated self-report mood scales: the Depression Anxiety Stress Scales (DASS) [8] and the Profile of Mood States (POMS) questionnaire [9]. The DASS was designed to measure three distinct but related negative affective states in nonclinical and research populations: depression (DASS-D) assesses dysphoria and anhedonia; anxiety (DASS-A) assesses autonomic arousal and subjective anxiety; and stress (DASS-S) assesses nervous arousal and agitation. The POMS (Educational and Industrial Testing Service, San Diego, CA) is one of the most widely used and accepted mood scales in healthy populations and estimates the intensity of mood disturbance covering six mood domains: tension-anxiety (POMS-T); depression-dejection (POMS-D); anger-hostility (POMS-A); fatigue-inertia (POMS-F); confusion-bewilderment (POMS-C); and vigor-activity (POMS-V).

At baseline, participants were randomized in block sizes of three to ensure equal balance in the following diet groups: omnivore (OMN), fish (FISH), or vegetarian (VEG). OMN participants were directed to continue consuming meat and/or poultry at least once daily. FISH participants were directed to avoid meat and poultry and consume at least 3-4 servings of seafood weekly (eggs were permitted). VEG participants were directed to avoid all animal foods except dairy for the 2-wk trial period. Participants were given written diet instructions and directed to maintain their activity pattern and lifestyle habits. The research staff also administered a brief computer-based cognitive test at baseline and at trial completion to mask our focus on mood. Participants were contacted at least twice during the study period to promote diet compliance. A brief survey assessing diet compliance and physical side effects was administered at trial completion.

Descriptive statistics were reported for population characteristics (mean ± SE); all outcome measures were presented as medians with interquartile ranges (IQR) as data were not normally distributed. The DASS data were normalized by removal of one outlier (from VEG group; data point was > 3SD from mean) and transformed by square root function. The transformed DASS data were analyzed using univariate ANOVA to compare 2-wk change scores between groups. For all other data, non-parametric analyses were performed using the Kruskal Wallis test to compare 2-wk change scores between groups. The Chi-square test and one-way ANOVA test were used to examine comparisons between groups at baseline. Spearman's correlation was used to assess relationships between variables. The Statistical Package for the Social Sciences (SPSS 17 for Windows, 2009, Chicago IL) was used for all analyses and *p *≤ 0.05 was considered significant.

## Results

All thirty-nine participants completed the 2-week trial and reported their diet compliance at > 95%; 82% of the participants were female. Groups did not differ at baseline by age, gender, BMI, educational level, ethnicity, total physical activity level or fatty acid intakes. There was a significant difference between the OMN and VEG groups for the POMS-C score at baseline [median (IQR): 9 (8) and 3 (5) respectively; *p *= .007].

After the 2-wk intervention period, dietary EPA, DHA, and AA fell to negligible amounts in VEG participants (*p *< .05), and the n-6 to n-3 ratio rose 60% in this group, as ALA declined (*p *< .05) (Table1). The intervention did not significantly impact the n-6 to n-3 ratio for OMN or FISH participants; however, dietary EPA/DHA in the FISH participants was increased 95-100% (*p *< .05) (Table 1). Dietary fatty acid intakes were unchanged in the OMN group. The data demonstrate that the intervention successfully manipulated levels of EPA/DHA in the diets of FISH and VEG participants. Although fatty acid tissue concentrations were not assessed, two weeks is adequate for significant changes to be observed [10].

**Table 1:** Dietary fatty acids at baseline and after the 2-wk diet intervention for participants randomized to omnivorous, fish, or vegetarian diets^a^

	Omnivorous diet		Fish diet		Vegetarian diet		
	Baseline	Week 2	Baseline	Week 2	Baseline	Week 2	P
α linolenic, g	0.37 (0.68)	0.44 (0.79)	0.79 (0.89)	0.28 (0.81)	0.58 (0.70)	0.38 (0.50)	0.874
Eicosa-pentaenoic, g	0.06 (0.10)	0.09 (0.12)	0.10 (0.13)	0.23 (0.26)^b^	0.06 (0.10)	0.00 (0.00)^bc^	.001
Docosa-hexaenoic, g	0.12 (0.15)	0.12 (0.17)	0.19 (0.25)	0.36 (0.37)^b^	0.09 (0.13)	0.00 (0.00)^bc^	.001
Total n-3, g	0.69 (0.88)	0.61 (0.85)	1.10 (0.74)	1.11 (1.48)	0.73 (0.60)	0.39 (0.50)	.065
Linolenic, g	4.29 (4.49)	3.54 (4.48)	6.49 (6.85)	5.53 (7.49)	4.63 (4.08)	4.69 (4.72)	.790
Arachidonic, g	0.12 (0.12)	0.08 (0.12)	0.14 (0.07)	0.08 (0.11)	0.10 (0.06)	0.00 (0.00)^bc^	.001
Total n-6, g	5.63 (3.78)	4.21 (4.49)	6.77 (6.95)	5.71 (7.11)	5.03 (4.64)	4.70 (5.05)	.821
Saturated, g	10.44 (7.92)	9.69 (10.22)	13.15 (9.86)	7.56 (8.72)	14.66 (12.36)	8.44 (9.99)	.173
Total fatty acids, g	29.78 (13.79)	30.12 (25.73)	39.52 (34.8)	30.64 (31.59)	38.40 (28.45)	22.86 (23.86)	.278
n-6/n-3*	7.32 (5.21)	7.50 (5.67)	6.76 (5.63)	3.99 (6.21)	7.88 (2.12)	14.55 (10.60)^bc^	.001

^a ^Data are median (IQR); P value for 2-wk change in scores by group (Kruskal Wallis test)

^b ^Change from baseline significantly greater for vegetarian diet than for omnivorous diet

^c ^Change from baseline significantly greater for vegetarian diet than for fish diet

* LA + AA/ALA + EPA + DHA

The 2-wk change (decline) in DASS-Stress scores was significantly greater in VEG participants compared to OMN and FISH participants suggesting greater improvement in this mood parameter for the VEG participants (Table2). The DASS-S scale measures a relatively narrow syndrome of "tension/stress" similar to the DSM-IV diagnosis of Generalized Anxiety Disorder [11]. These data suggest that individuals who eliminate meat, fish, and poultry may cope better with mental stress than omnivores. The 2-wk change in POMS-C scores was also significantly greater for VEG participants compared to OMN and FISH participants, and POMS-Tension and POMS-Total scores tended to be greater for VEG participants (declines). POMS-C and POMS-T subscales (along with POMS-D) are highly correlated and in combination represent psychological distress [12]. There were no significant changes in other POMS scores (Table 2).

**Table 2:** DASS and POMS scores at baseline and after the 2-wk diet intervention for participants randomized to omnivorous, fish, or vegetarian diets^a^

	Omnivorous diet		Fish diet		Vegetarian diet		
	Baseline	Week 2	Baseline	Week 2	Baseline	Week 2	P
DASS-global	7 (13)	6 (13)	13 (16)	6 (12)	11 (12)	4.59 (9)	.559
DASS-D *depression*	1 (2)	1 (2)	2 (17)	1 (4)	1 (4)	1 (2)	.984
DASS-A *anxiety*	25 (60)	10 (19)	27 (37)	13 (15)	55 (66)	15 (32)	.502
DASS-S *stress*	20 (28)	14 (16)	18 (18)	8 (13)	21.5 (25)	8.5 (14)^b^	.045
POMS-Total	8 (26)	3 (22)	18 (18)	8 (13)	21.5 (25)	5 (23)	.087
POMS-T *tension*	6 (4)	5 (5)	7 (5)	7 (4)	8 (8)	4(4)	.061
POMS-D *depression*	3 (5)	3 (4)	5 (6)	2 (7)	3 (10)	2 (2)	.448
POMS-A *anxiety*	3 (16)	3 (5)	5 (10)	4 (4)	6 (11)	2 (5)	.713
POMS-F *fatigue*	5 (5)	4 (6)	7 (5)	4 (5)	8 (7)	3 (9)	.936
POMS-C *confusion*	3 (5)	4 (5)	5 (5)	4 (5)	9 (8)	3 (6)^b^	.003
POMS-V *vigor*	17 (9)	18 (9)	19 (10)	14 (9)	14 (14)	19 (13)	.729

^a ^Data are median (IQR); P value for 2-wk change in scores by group (Oneway ANOVA for DASS data which were square root transformed to achieve normality; Kruskal Wallis test for POMS data which were not normally distributed)

^b ^Change from baseline significantly greater for vegetarian diet than for omnivorous or fish diet (between group analysis)

## Discussion

These results support the results of our cross-sectional study which found that vegetarians reported significantly better mood than their non-vegetarian counterparts [6]. Although omnivores who consumed fish frequently and avoided meat and poultry for two weeks did not significantly improve their mood, those who adopted a lacto vegetarian diet did improve their mood. These data suggest that consuming a diet high in meat, fish, and poultry may negatively impact mental state. Beyond differences in the ratio of long-chain fatty acids, vegetarian diets are typically rich in antioxidants, potentially conveying mood protection for the VEG group via reduction of oxidative stress [13].

Early human evolution is theorized to have coincided with increasing reliance on animal source foods such as wild game and seafood [14], however, the average n-6 to n-3 fatty acid profile of modern grain-fed meat is 5X higher than grass-fed meat, a product similar to the wild game in our hunter-gatherer diet [15,16]. The amount of meat and poultry consumed is important since very little AA is formed from LA [17]; in fact, diets high in short chain essential fatty acids down regulate conversion to longer chain metabolites, particularly AA, and experimental diets high in LA do not raise tissue AA [18,19]. Preformed AA, however, is readily incorporated into tissues and competes for desaturases with EPA, increasing production of proinflammatory metabolites such as PGE_2 _and TNFa [17]. This raises the risk for inflammation-based chronic diseases including depression since these metabolites are associated with altering mood-regulating mechanisms [20].

Participants in the FISH group consumed a relatively high fish intake (~270-364 mg/d of EPA/DHA vs the U.S. mean daily intake of ~88 mg/d) [21]. Long-chain n-3 fatty acids incorporate steadily into brain phospholipids with a reciprocal decrease in n-6 fatty acid content [22]. Numerous studies show mood protective effects of high fish intakes in nonvegetarian populations [23], however, mood scores in our FISH participants did not improve significantly within the study time frame.

Failure to monitor weight of the participants beyond the baseline BMI calculation was a study limitation, possibly missing weight fluctuations that could confound mood. Also, in light of the results, measures of blood fatty acid concentrations may have shown that long-chain n-3 intake and status in the vegetarians was not as disparate as expected, as observed in the recent EPIC-Norfolk study [24]. Measures of blood inflammatory markers associated with mood would also have strengthened results.

## Conclusions

Our results suggest that reducing meat, fish, and poultry may improve some domains of short-term mood state in modern omnivores. Exploring this phenomenon further is warranted, as reductions in dietary meat, fish, and poultry would not only reduce health risks but could benefit the environment as well.

## Competing interests

The authors declare that they have no competing interests.

## Authors' contributions

BLB participated in the study design and acquisition of data, performed the statistical analysis and interpretation of results, and drafted the manuscript. CSJ participated in the study design and acquisition of data, assisted in the statistical analysis and interpretation of results, and edited the manuscript. Both authors read and approved the final manuscript.
